# Fission Yeast Rad52 Phosphorylation Restrains Error Prone Recombination Pathways

**DOI:** 10.1371/journal.pone.0095788

**Published:** 2014-04-18

**Authors:** Angela Bellini, Pierre-Marie Girard, Ludovic Tessier, Evelyne Sage, Stefania Francesconi

**Affiliations:** 1 Institut Curie, Centre de Recherche, Orsay, France; 2 CNRS UMR 3348, Bât. 110, Centre Universitaire, Orsay, France; Universita’ di Milano, Italy

## Abstract

Rad52 is a key protein in homologous recombination (HR), a DNA repair pathway dedicated to double strand breaks and recovery of blocked or collapsed replication forks. Rad52 allows Rad51 loading on single strand DNA, an event required for strand invasion and D-loop formation. In addition, Rad52 functions also in Rad51 independent pathways because of its ability to promote single strand annealing (SSA) that leads to loss of genetic material and to promote D-loops formation that are cleaved by Mus81 endonuclease. We have previously reported that fission yeast Rad52 is phosphorylated in a Sty1 dependent manner upon oxidative stress and in cells where the early step of HR is impaired because of lack of Rad51. Here we show that Rad52 is also constitutively phosphorylated in *mus81* null cells and that Sty1 partially impinges on such phosphorylation. As upon oxidative stress, the Rad52 phosphorylation in *rad51* and *mus81* null cells appears to be independent of Tel1, Rad3 and Cdc2. Most importantly, we show that mutating serine 365 to glycine (S365G) in Rad52 leads to loss of the constitutive Rad52 phosphorylation observed in cells lacking Rad51 and to partial loss of Rad52 phosphorylation in cells lacking Mus81. Contrariwise, phosphorylation of Rad52-S365G protein is not affected upon oxidative stress. These results indicate that different Rad52 residues are phosphorylated in a Sty1 dependent manner in response to these distinct situations. Analysis of spontaneous HR at direct repeats shows that mutating serine 365 leads to an increase in spontaneous deletion-type recombinants issued from mitotic recombination that are Mus81 dependent. In addition, the recombination rate in the *rad52-S365G* mutant is further increased by hydroxyurea, a drug to which mutant cells are sensitive.

## Introduction

Eukaryotic cells are continuously challenged by both endogenous and exogenous insults that threaten genome integrity. In order to prevent genome instability, complex DNA damage response and repair pathways dedicated to specific lesions have evolved [1], [2].

Homologous recombination (HR) is one of these pathways specifically required for double-strand DNA breaks (DSBs), single-strand DNA gap and interstrand crosslinks repair, as well as for recovery of blocked or collapsed replication forks. Failure to properly execute HR is associated with different human genetic syndromes and tumor progression [3], [4], [5], [6]. Fundamental to this pathway is the presence of a donor homologous sequence that serves as a template to restore the information at the damaged site.

In mitotic cells, repair of DSBs by HR is believed to occur mainly according to the SDSA (Synthesis-Dependent Strand Annealing) model that leads to non-crossover products thus preventing loss of heterozygosity and chromosomal translocations [7]. After resection at DSB, a 3′ single strand DNA tail is generated that will be first coated by RPA protein. Then, mediator proteins, including Rad52, will help in replacing RPA by the recombinase Rad51, allowing formation of a Rad51 nucleofilament able to engage in strand invasion and D-loop formation. Following DNA synthesis using the homologous unbroken DNA sequence as a template, the D-loop is displaced and newly synthesized DNA pairs to the other end of the break. When the homologous sequences are heteroallelic, the paired DNA will contain a mismatch recognized by the mismatch repair system (MMR) that will either reject the invading strand or correct the mismatch possibly leading to gene conversion [4]. However, when the D-loop is not displaced, four-way DNA structures named Holliday junctions (HJ) are formed and either resolved by the action of specific helicases and topoisomerases activities, or are processed by specialized endonucleases including Mus81 [8], [9]. When the DSB is flanked by sequence repeats, it can be repaired by the single-strand annealing (SSA) pathway that does not involve strand invasion and D-loop formation and thus is Rad51 independent, but Rad52 dependent. This mechanism results in loss of genetic material between the repeats. Rad52 protein from *Schizosaccharomyces pombe* is also required for microhomology-mediated end-joining (MMEJ) that is a type of SSA involving short repeat sequences [10]. Furthermore, fission yeast Rad52, similarly to its human homologue, can promote strand invasion in a Rad51 independent manner and therefore formation of a D-loop that can be cleaved by the Mus81 structure-selective endonuclease [8], [11].

In a variety of organisms, the Mus81 endonuclease appears to play a key role in processing different DNA intermediates resulting from induced or spontaneous replication fork perturbation and in the HR mechanism at broken replication forks [12], [13], [14]. However, during fork perturbation Mus81 activity is kept under control by the replication checkpoint to avoid excessive DNA fragmentation [15]. In the same line, Mus81 endonuclease activity is required for survival of thermosensitive alleles of fission yeast replicative DNA polymerases and of human DNA replication checkpoint deficient cells, although both situations lead to increased genomic instability [14], [16].

During DNA replication, a single-ended DSB can occur at broken replication forks and it will be repaired by the HR machinery through a mechanism known as break-induced replication (BIR) that requires both Rad51 and Rad52 proteins [17]. Differently, HR-dependent fork restart can occur independently of DSB formation and relies on template switch and homology-driven template exchange mechanisms, both requiring Rad52 and Rad51 functions. In addition, a second homology-driven template exchange mechanism that requires only Rad52 has been proposed [18]. However, while HR helps to complete genome replication by restarting broken forks or by overcoming replication fork barriers, it is not harmless and promotes chromosome rearrangements [6], [19].

The Rad52 protein, which is conserved in all eukaryotes, acts as an oligomeric ring that can interact with ssDNA, RPA and Rad51. Sequence analysis of Rad52 homologues from different organisms shows that the N-terminal half of the protein, that comprises the DNA binding domain, is highly conserved while the C-terminal part is less conserved. The Rad51 interaction domain has been mapped between amino acids 409 and 412 in Rad52 from *Saccharomyces cerevisiae* [20] and between amino acids 290 and 330 in human Rad52 [21]. In fission yeast, the C-terminal region of Rad52 extending from amino acid 310 has been shown to be required for Rad51 interaction by two hybrid system [22]. While it is clear that in both budding and fission yeasts Rad52 mediates Rad51 recombination function, in others eukaryotes this function is mainly performed by BRCA2 protein, that is absent in yeast. However, human Rad52, but not BRCA2, retains the ability to promote SSA. In addition, mammalian Rad52 becomes important in cells carrying BRCA2 mutations since its inactivation displays synthetic lethality [23], [24], [25].

Rad52 undergoes different post-translational modifications such as sumoylation and phosphorylation, but the outcomes of these modifications differ among the organisms [26]. For example, Rad52 from *S. cerevisiae* is constitutively phosphorylated and additional phosphorylation occurs at beginning of S phase but not upon exposure to DNA damaging agents [27]. In contrast, human Rad52 is phosphorylated at Y104 upon DNA damage by the c-Abl kinase in an ATM and DNA-PKcs dependent manner leading to increased SSA activity [28].

*S. pombe* Rad52 protein functions in both Rad51 dependent and independent pathways of HR [11], [22] and it has been shown to undergo SUMO modification [29]. We have previously reported that fission yeast Rad52 is phosphorylated in cells experiencing oxidative stress and in cells lacking Rad51 recombinase. In both cases, phosphorylation was abolished by Sty1 depletion, the major stress responsive MAPK of fission yeast [30].

Here we show that Rad52 phosphorylation occurs also in *mus81* null cells and that mutating serine 365 to glycine results in loss of Rad52 phosphorylation in *rad51* and *mus81* null cells and increases spontaneous deletion type mitotic recombinants occurring at direct repeats that are Mus81 dependent.

## Materials and Methods

### Yeast Strains, Media, Growth Conditions

Strains used in this study are listed in Table 1. Strains were grown in YE-rich medium (DIFCO) containing 2% glucose and supplemented with adenine, leucine, uracile, arginine and histidine [31]. Caffeine (Sigma) treatment was performed with 10 mM caffeine for 2 hours. Synchronization in early S phase was achieved by 4 hours treatment with 12 mM hydroxyurea (HU) (Sigma) and then cells were collected and released in fresh medium. Treatment with UVA radiation or H_2_O_2_ of synchronized cells was performed as described in [30].

**Table 1:** strains used in this study.

STRAIN	GENOTYPE	SOURCE
*rad52+*	*h−*	Laboratory collection
*rad52YFP*	*h− rad52YFP:KanR*	[32]
*rad52-S365G-YFP*	*h− rad52-S365G-YFP:KanR*	This study
*rad51-d rad52YFP*	*h+ rad51::ura4+ ura4-D18 rad22YFP:KanR*	[30]
*rad51-d rad52-S365G-YFP*	*h− r rad51::ura4, rad22-S365G-YFP:KanR ura4-D18*	This study
*rad51-d sty1-d rad52YFP*	*h+ rad51::ura4+ sty::ura4+ ura4-D18 rad52YFP:KanR leu1-32*	[30]
*rad51-d tel1-d rad52YFP*	*h+ rad51::ura4+ tel1::KanR rad22YFP:KanR ura4-D18 leu1-32*	This study
*rad51-d cdc2-33ts rad52YFP*	*h− rad51::ura4+cdc2-33ts rad22YFP:KanR ura4-D18*	This study
*mus81-d rad52YFP*	*h− mus81::KanR ura4-D18 rad22YFP:KanR*	This study
*mus81-d sty1-d rad52YFP*	*h+ mus81::KanR sty1::ura4+ ura4-D18 rad22YFP:KanR*	This study
*mus81-d rad52-S365G-YFP*	*h+ mus81::KanR ura4-D18 rad52-S365G-YFP:KanR*	This study
*mus81-d rad3-d rad52YFP*	*h+ mus81::KanR rad3::ura4+ ura4-D18 leu1-32 rad22YFP:KanR*	This study
*rad52-d*	*smt0 rad52::ura4+ ura4-D18*	Laboratory collection
*rad3-d*	*h− leu1-32 ura4-D18 rad3::ura4+*	Laboratory collection
*rad51-d rad52-d*	*h− smt0 rad52::ura4+ rad51::ura4+ ura4-D18*	Laboratory collection
*mus81-d rad52-d*	*h− smt0 rad52::ura4 ura4-D18 mus81::KanR*	Laboratory collection
*sty1-GFP*	*h90 sty1::sty1-GFP-HA-KanR ade6-M210 leu1-32 lys1-131 ura4-D18*	YGRC
*rad51-d sty1-GFP*	*h− rad51::ura4+ ura4-D18 sty1::sty1-GFP-HA-KanR ade6-M216 leu1-32*	This study
*mus81-d sty1-GFP*	*h− mus81::ura4+ ura4-D18 sty1::sty1-GFP-HA-KanR ade6-M210 leu1-32*	This study
*HRS rad52YFP*	*h+ rad52YFP:KanR ura4-D18 leu1-32 his3-D1 arg3-D4 ade6-L469/pUC8/his3+/ade6-M375*	This study
*HRS rad52-S365G-YFP*	*h+ rad52-S365G-YFP:KanR ura4-D18 leu1-32 his3-D1 arg3-D4 ade6-L469/pUC8/his3+/ade6-M375*	This study
*HRS mus81-d rad52YFP*	*h+ mus81::KanR rad52YFP:KanR ura4-D18 leu1-32 his3-D1 arg3-D4 ade6-L469/pUC8/his3+/ade6-M375*	This study
*HRS mus8-d rad52-S365G-YFP*	*h+ mus81::KanR rad52-S365G-YFP:KanR ura4-D18 leu1-32 his3-D1 arg3-D4 ade6-L469/pUC8/his3+/ade6-M375*	This study

*rad52-S365G-YFP* strain was constructed using a plasmid containing about 700 bp of C-terminal sequence of *rad52^+^* fused in frame with YFP encoding sequence and a kanamycin resistance (*KanR*) selectable marker as described in [32]. Plasmid was used for site directed mutagenesis to modify codon AGC encoding Ser365 to codon GGC encoding a Glycine using the QuickChange Site-Directed Mutagenesis Kit (Stratagene). Oligonucleotides were: (5′-CTTTAATCCTCGTCTCGACGGCCCTTCTATTAGG-3′) and (5′-CCTAATAGAAGGGCCGTCGAGACGAGGATTAAAG-3′). The mutagenized plasmid was checked by sequencing. After linearization at AflII restriction site, the plasmid was integrated at *rad52^+^* locus by transforming strain 972 h-. Transformants were selected for G418 (Sigma-Aldrich) resistance after 24 hours of growth on YE-rich medium. Correct integration was checked by colony PCR using appropriated oligonucleotides. The presence of mutation was confirmed by sequencing the PCR amplified allele. Standard genetic techniques were used for construction of all other strains.

### SDS-PAGE and Immunoblot

Protein extracts were done according to [33]. To analyze Rad52YFP, 80 µg of each protein extract was separated by electrophoresis at 40 Volts over-night on 7.5% acrylamide SDS-PAGE (acrylamide:bis-acrylamide 37.5∶1) using the STURDIER vertical SE 400 gel unit (Hoefer Scientific Instruments). Proteins were transferred for 2 hours at 120 Volts on nitrocellulose membrane (PROTRAN Whatman) using a Biorad Trans-Blot Cell system. Membranes were probed with mouse anti-GFP antibody (Roche).

### Microscopy and Flow Cytometry

Nuclei were visualized by DAPI (4′-6′-diamino-2-phenylindole, SIGMA) staining of cells fixed in 70% ethanol. Pictures were taken using a Leica Microsystems DMRD Microscope with a 100x oil immersion objective and a Princeton CoolSnap fx cooled CCD camera setting the binning at 1. For observation of living cells expressing Rad52YFP or Sty1GFP protein the exposure time was of 5 s and binning was set at 2. Image capture software was MetaView (Universal Imaging) whereas image processing software was ImageJ.

DNA content was analyzed by staining fixed cells with sytox green (Invitrogen) followed by flow cytometry analysis with FACSCalibur flow cytometer (Becton Dickinson). Data were plotted using CellQuest software.

### Genotoxic Treatments

Genotoxic sensitivity of strains was tested by drop assay. Cells were grown in YE-rich medium, diluted to 1.3×10^6^ cells/ml and 7.5 µl of sequential four-fold dilutions were spotted on the appropriate plates. To determine UVC and gamma ray sensitivities, plates were irradiated with a 254 nm light source in a Stratalinker (Stratagene) or with a Cs^137^ gamma source at a dose rate of 0.16 Gy/s, respectively. Plates were incubated at 30°C for 4–5 days and then photographed.

### Fluctuation Test and HR Rate Estimation

Fluctuation test was done as follow: 9 Ade− His+ colonies were independently inoculated in 10 ml of YE-rich medium and incubated at 30°C with agitation till saturation. Cells were plated on YE-rich medium to estimate viability. About 1.8×10^6^ viable cells for each culture were plated on EMM medium lacking adenine to estimate the frequency of Ade+ recombinants. Plates were replicated on EMM lacking adenine and histidine to estimate frequency of Ade+ His+ recombinants. Frequencies were analyzed by MSS-MLE (Ma-Sandri-Sarkar Maximum Likelihood Estimator) method with the program FALCOR (Fluctuation AnaLysis CalculatOR) to estimate the rate of recombination [34]. For each strain at least three independent experiments of nine cultures each were performed.

To establish the HU induced frequency of recombinants, single Ade− His+ colonies were streaked on YE plates containing or not 5 mM HU. From these plates 9 independent clones were resuspended in water and cells were plated on YE-rich to establish survival and on selective medium to obtain the Ade+ recombinants. Two independent experiments were performed.

## Results

### Mutation at S365 of Rad52 Prevents Rad52 Phosphorylation in *rad51* and *mus81* Null Cells

Through *in silico* analysis (KinasePhos, NetPhos and GPS softwares), we identified several possible phosphorylation sites in fission yeast Rad52 protein. We concentrated on two possible phosphorylatable sites by MAPK located at the C-terminal of the protein: Threonine 333 (T333) and Serine 365 (S365). By site directed mutagenesis, we mutated T333 to A and S365 to G and the mutated sequences were integrated at the genomic locus as described in material and methods.

**Figure 1:**
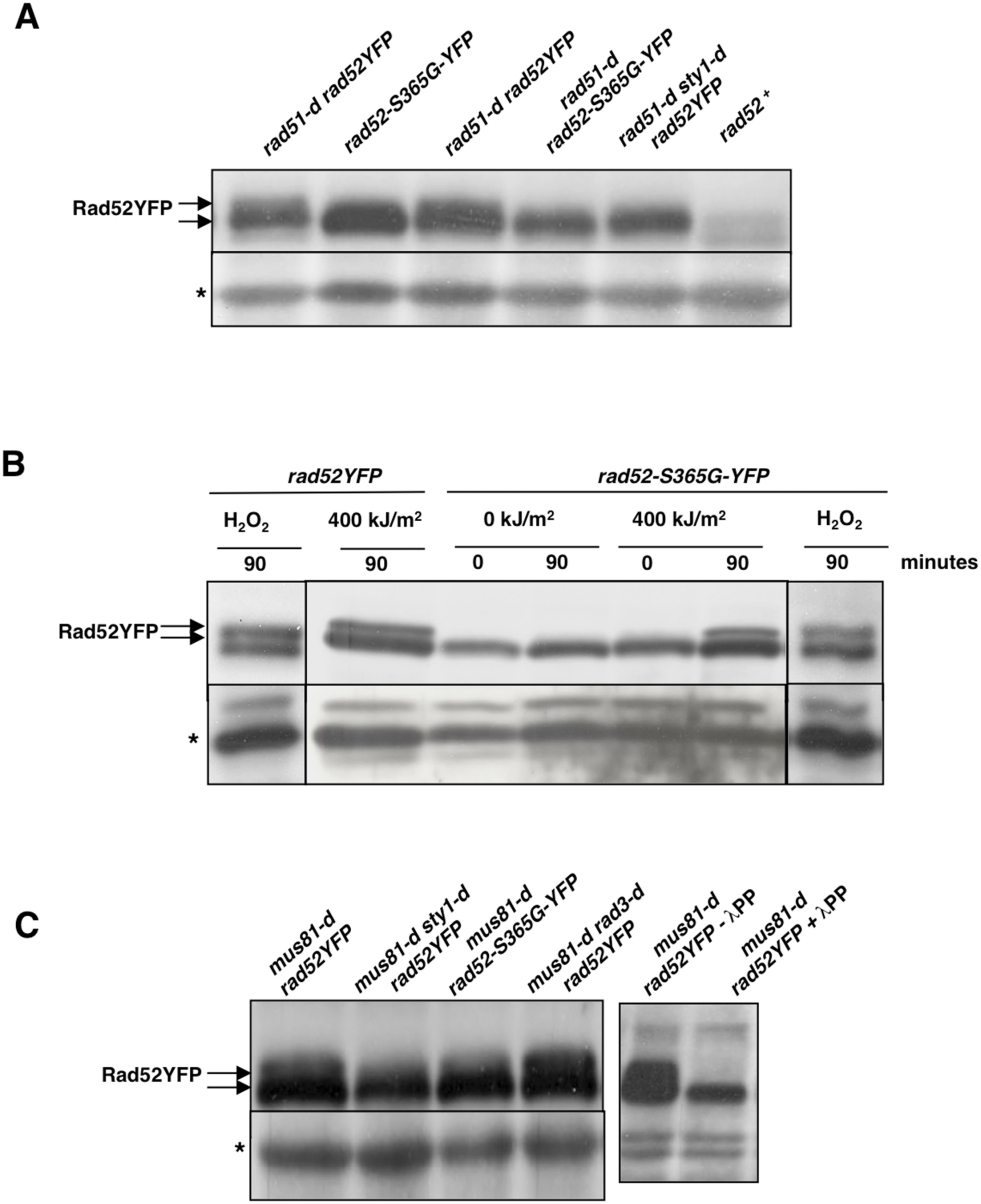
Mutating Ser 365 to Gly in Rad52 abrogates Rad52 phosphorylation in *rad51* and *mus81* null cells but not in cells experiencing oxidative stress. (**A**) Immunoblot with anti-GFP antibodies of protein extracts from the indicated strains reveals loss of Rad52 phosphorylation *in rad52-S365G-YFP* mutant and in *sty1-d* cells. Star indicates aspecific band serving as loading control. (**B**) Immunoblot with anti-GFP antibodies of protein extracts from strains *rad52YFP* and *rad52-S365G-YFP* synchronized by HU and released into cell cycle for 0 or 90 minutes either after UVA irradiation or in the presence of 250 µM H_2_O_2_. Star indicates aspecific band serving as loading control. (**C**) Immunoblot with anti-GFP antibodies of protein extracts from the indicated strains. Left panel shows Rad52 phosphorylation in *mus81-d* cells that is Sty1 dependent, serine 365 dependent but Rad3 independent. Right panel shows treatment with **l** phosphatase (+**l** PP) of protein extracts from *mus81-d rad52YFP* cells. Star indicates aspecific band serving as loading control.

We were unable by different strategies to obtain stable haploid or diploid strains expressing Rad52 mutated at T333, suggesting that this mutation is highly deleterious for cell survival and might act as a dominant negative. A haploid strain expressing at the endogenous locus the mutated Rad52-S365G protein tagged with YFP (*rad52-S365G-YFP*) was obtained. The *rad52-S365G-YFP* allele was then crossed into cells lacking Rad51 (*rad51-d rad52-S365G-YFP*). Rad52 protein expressed in strains *rad52-S365G-YFP*, *rad51-d rad52YFP*, *rad51-d rad52-S365G-YFP, rad51-d sty1-d rad52YFP* was analyzed by western blot using anti-GFP antibodies. A strain expressing untagged Rad52 protein (*rad52^+^*) was used as a negative control. As previously published, Rad52 phosphorylation was observed in *rad51-d* cells but not in *rad51-d sty1-d* cells (Figure 1A). Both *sty1* deletion and mutation of S365 to G abolished Rad52 phosphorylation in *rad51-d* cells (Figure 1A).

Because we have shown that Rad52 undergoes a Sty1 dependent phosphorylation when cells experience oxidative stress [30], we asked if mutating S365 would also abolish Rad52 phosphorylation under these conditions. Thus, *rad52YFP* and *rad52-S365G-YFP* cells were exposed to UVA or H_2_O_2_ after synchronization in early S phase as described in [30]. Rad52 phosphorylation was assessed by western blotting at 0 and 90 minutes after irradiation and after 90 minutes of release in medium containing H_2_O_2_. As shown in figure 1B, the Rad52-S365G-YFP protein was phosphorylated to the same extent than Rad52YFP wild type protein following both treatments.

Because fission yeast Mus81 plays an important role together with Rad52 in a Rad51 independent pathway of HR, we assessed the phosphorylation state of Rad52 in *mus81* null cells. We found that Rad52 is also phosphorylated in this genetic background as shown by the presence of a band with slower mobility that was eliminated by treatment with **l** phosphatase (Figure 1C). More importantly, Rad52 phosphorylation was partially abolished by knocking out the *sty1* gene or by mutating Rad52 at serine 365 (Figure 1C). On the opposite, knock out of the *rad3* gene did not impact on Rad52 phosphorylation in cells lacking Mus81.

These experiments indicate that S365 contributes to Rad52 phosphorylation *in vivo* in cells lacking Rad51 or Mus81 but not in cells experiencing oxidative stress, although Rad52 phosphorylation is dependent on intact MAPK pathway in all situations. Thus, Rad52 seems to undergo phosphorylation at different sites.

### Tel1, Rad3 and Cdc2 are unlikely to be the Kinases Required for Rad52 Modification in *rad51* Null Cells

**Figure 2:**
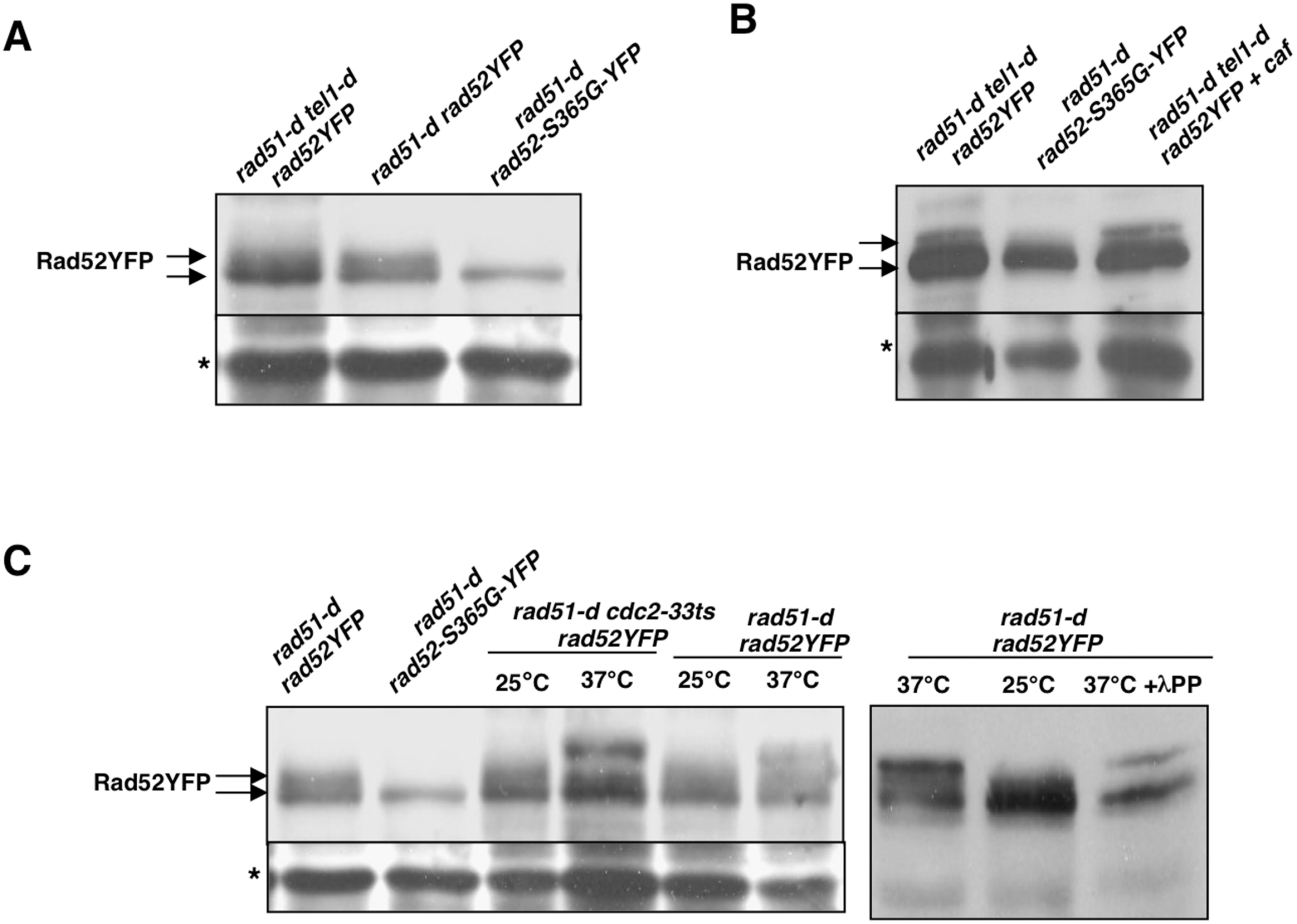
Tel1, Rad3 and Cdc2 kinases are dispensable for Rad52 phoshorylation in *rad51-d* background. (**A**) Immunoblot with anti-GFP antibodies of protein extracts from the indicated strains reveals Rad52 phosphorylation in cells lacking Rad51 and Tel1 kinase. (**B**) Inhibiting Rad3 by caffeine (+ caf) treatment does not abrogate Rad52 phosphorylation in *rad51-d tel1-d* cells. (**C**) Immunoblot with anti-GFP antibodies of protein extracts from the indicated strains reveals Rad52 phosphorylation in cells lacking the Cdc2 kinase. Star indicates aspecific band serving as loading control. **l** phosphatase (+**l** PP) treatment does not reverse the slower migrating band detected in extracts from cells grown at 37°C.

Because we have shown that under oxidative stress the Rad52 phosphorylation occurs in the absence of either Rad3 (ATR), Tel1 (ATM) or Cdc2 kinases, we asked if these kinases might be involved in Rad52 phosphorylation in cells lacking Rad51. While we could not test Rad3 kinase in this genetic background, because the *rad51-d rad3-d* double mutant has a severe growth defect [35], we addressed the question for Tel1 and Cdc2 kinases and we use caffeine to inhibit Rad3. As shown in Figure 2A, strain *rad51-d tel1-d rad52-YFP* still displayed Rad52 phosphorylation. In addition, treating *rad51-d tel1-d* cells with caffeine (+ caf) in order to inhibit the caffeine sensitive Rad3 kinase, did not abolish the Rad52 phosphorylation (Figure 2B).

To test the implication of Cdc2 kinase we took advantage of the *cdc2-33* thermosensitive allele. Strains *rad51-d cdc2-33ts rad52YFP* and *rad51-d rad52YFP* were grown at the permissive temperature of 25°C and then shifted for two hours at the non-permissive temperature of 37°C. Rad52 phosphorylation was analyzed by western blot under permissive and non-permissive conditions for both strains. As shown in Figure 2C (left panel), both strains displayed Rad52 phosphorylation at 25°C as well as at 37°C. It is to note that under non-permissive conditions an additional slowing migrating band is detected in both strains, as if at 37°C further modifications of Rad52 protein occurred. However, treatment with **l** phosphatase of protein extracts from cells grown at 37°C didn’t reverse the slowest migrating band (Figure 2C right panel), indicating that it is unlikely to be a phosphorylated form of Rad52YFP.

These results indicate that Tel1, Rad3 and Cdc2 are unlikely to be the kinases required for Rad52 modification in *rad51* null cells.

### *rad52-S365G-YFP* Cells are HU Sensitive

**Figure 3:**
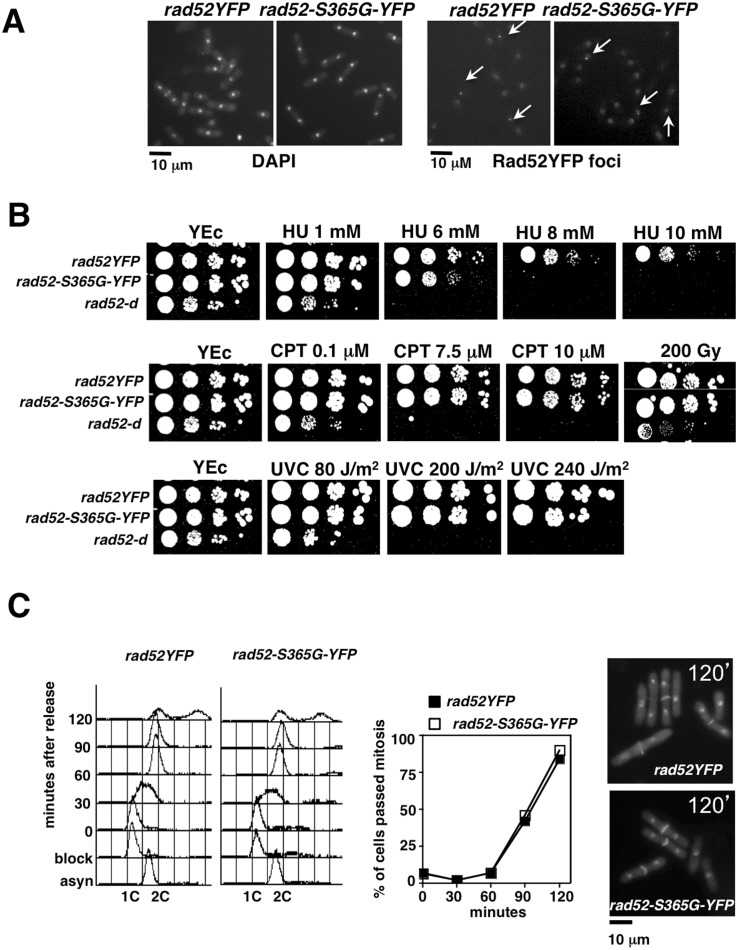
Mutation at Ser 365 results in sensitivity to chronic exposure to HU that is not due to checkpoint failure. (**A**) Microphotographs of cells from asynchronous cultures of the indicated strains stained with DAPI (left) and expressing wild type or mutated Rad52 protein tagged with YFP (right). Arrows indicates cells with visible Rad52 foci. (**B**) Drop assay to test DNA damage sensitivity of *rad52-S365G-YFP* mutant compared to wild type *rad52YFP* and *rad52-d* null cells. (**C**) Comparison of checkpoint function after release from HU block between wild type *rad52YFP* and mutant *rad52-S365G-YFP*: FACS analysis (left), percentage of cells with 2 nuclei (middle) and DAPI/calcofluor staining of cells at 120 minutes after release.

We first characterized *rad52-S365G-YFP* cells with respect to morphology, presence of Rad52YFP foci and response to different DNA damaging agents. As shown in Figure 3A, *rad52-S365G-YFP* cells stained with DAPI were indistinguishable from wild type *rad52YFP* cells. In addition, some *rad52-S365G-YFP* cells displayed Rad52 foci to the same extent as control strain *rad52YFP* (Figure 3A).

Then, the sensitivity to HU, CPT, γ-rays and UVC irradiation was tested by spot assay comparing *rad52-S365G-YFP* cells to control strains *rad52YFP* and *rad52-d*. As shown in Figure 3B, the *rad52-S365G-YFP* S365G allele conferred sensitivity only to chronic exposure to HU at concentrations higher than 6 mM.

We then tested if sensitivity to HU might be linked to a defect in checkpoint function. Wild type and mutant cells were synchronized in early S phase by HU and then released into cell cycle. Cell cycle progression was followed by flow cytometry analysis and by monitoring cells that passed mitosis (2 nuclei). Both strains progressed through cell cycle with the same kinetics and none of these strains showed the presence of abnormal mitosis, indicating that checkpoint function is retained (Figure 3C).

In addition, we have previously shown that Rad52 is not phosphorylated when cells are exposed to 12 mM HU where the replication checkpoint is fully activated or when cells are released from the HU block [30]. In conclusion, *rad52-S365G-YFP* sensitivity to HU is not linked to checkpoint failure or to lack of HU induced phosphorylation.

### *rad52-S365G-YFP* Allele doesn’t Change the Phenotype of either *rad51* or *mus81* Null Cells

As for *rad51+ mus81+* wild-type background (Figure 3A), the *rad52-S365G-YFP* allele didn’t change the cell morphology or Rad52 foci formation and frequency characteristic of *rad51-d* or *mus81-d* cells (data not shown) [32], [36].

**Figure 4:**
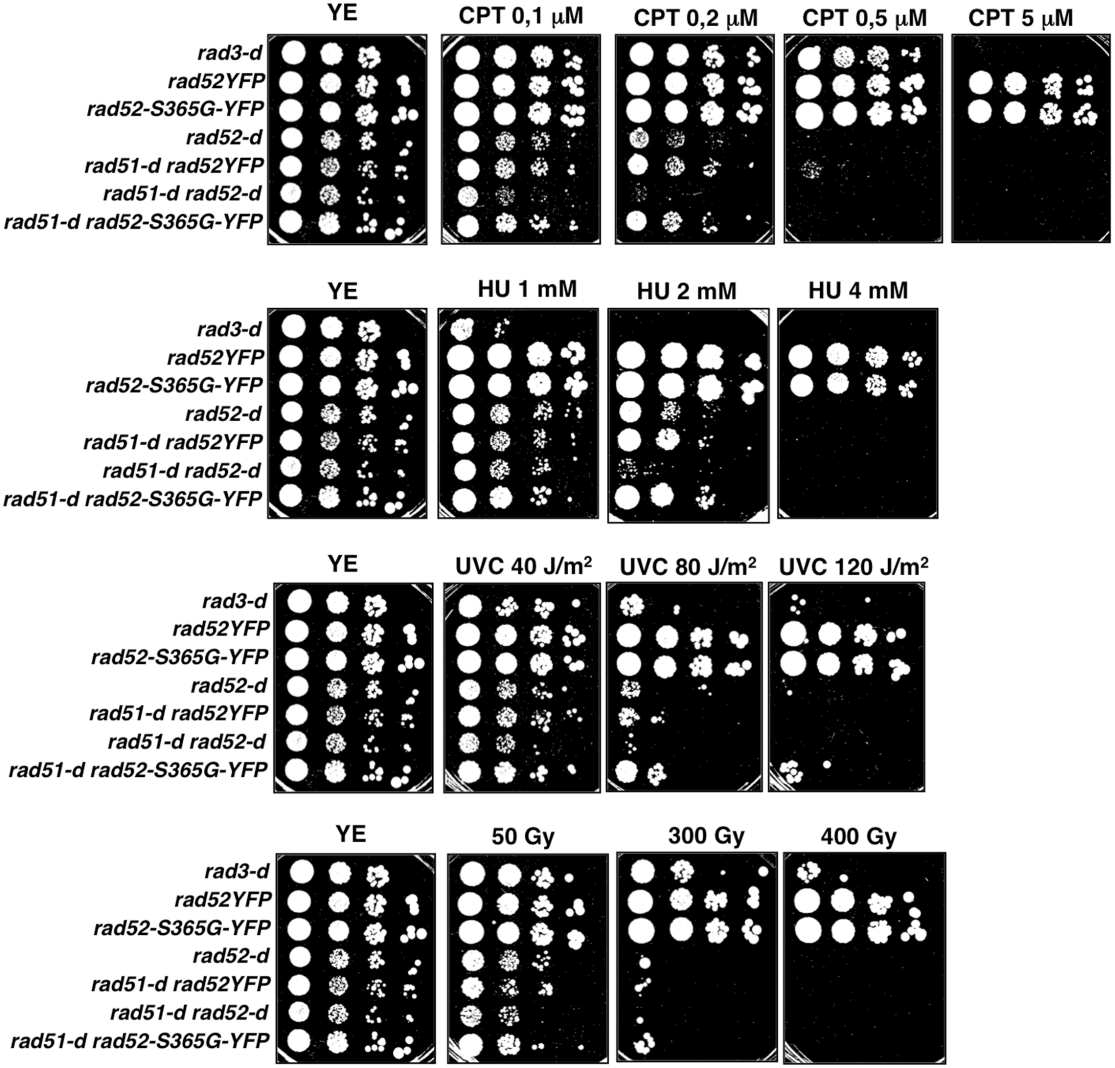
*rad52-S365G-YFP* allele doesn’t change the sensitivity of *rad51-d* cells to genotoxic treatments. Drop assay of the indicated strains on YEc medium. Plates were incubated 5 days at 30°C.

It has been reported that cells deleted for *rad52* (*rad52-d*) are more sensitive to genotoxins, with the exception for gamma irradiation, than cells lacking Rad51 (*rad51-d*) since both Rad51 dependent and independent pathways are impaired, and that the double mutant *rad52-d rad51-d* is slightly more sensitive to genotoxins than each single mutant [11]. Therefore, and to get insight into the biological function of S365, we analyzed the relationship of *rad52-S365G-YFP* allele to the *rad51* epistasis group. To do so, we compared the sensitivity to various DNA damaging agents of *rad51-d rad52-S365G-YFP* cells with that of *rad52-S365G-YFP*, *rad51-d rad52YFP*, *rad52-d* and *rad51-d rad52-d.* Strain *rad3-d,* lacking the central DNA damage checkpoint kinase, was used as a control. As shown in Figure 4, the response to genotoxins of strain *rad51-d rad52-S365G-YFP* was similar to that of single mutant *rad51-d rad52YFP*. This was also true for the HU response suggesting that the *rad52-S365G-YFP* allele, that is HU sensitive, might act in the Rad51 pathway. However, by this assay it is difficult to conclude in favor of an epistatic relationship because *rad51-d* cells are highly sensitive to 2 and 4 mM HU while *rad52-S365G-YFP* cells are not.

**Figure 5:**
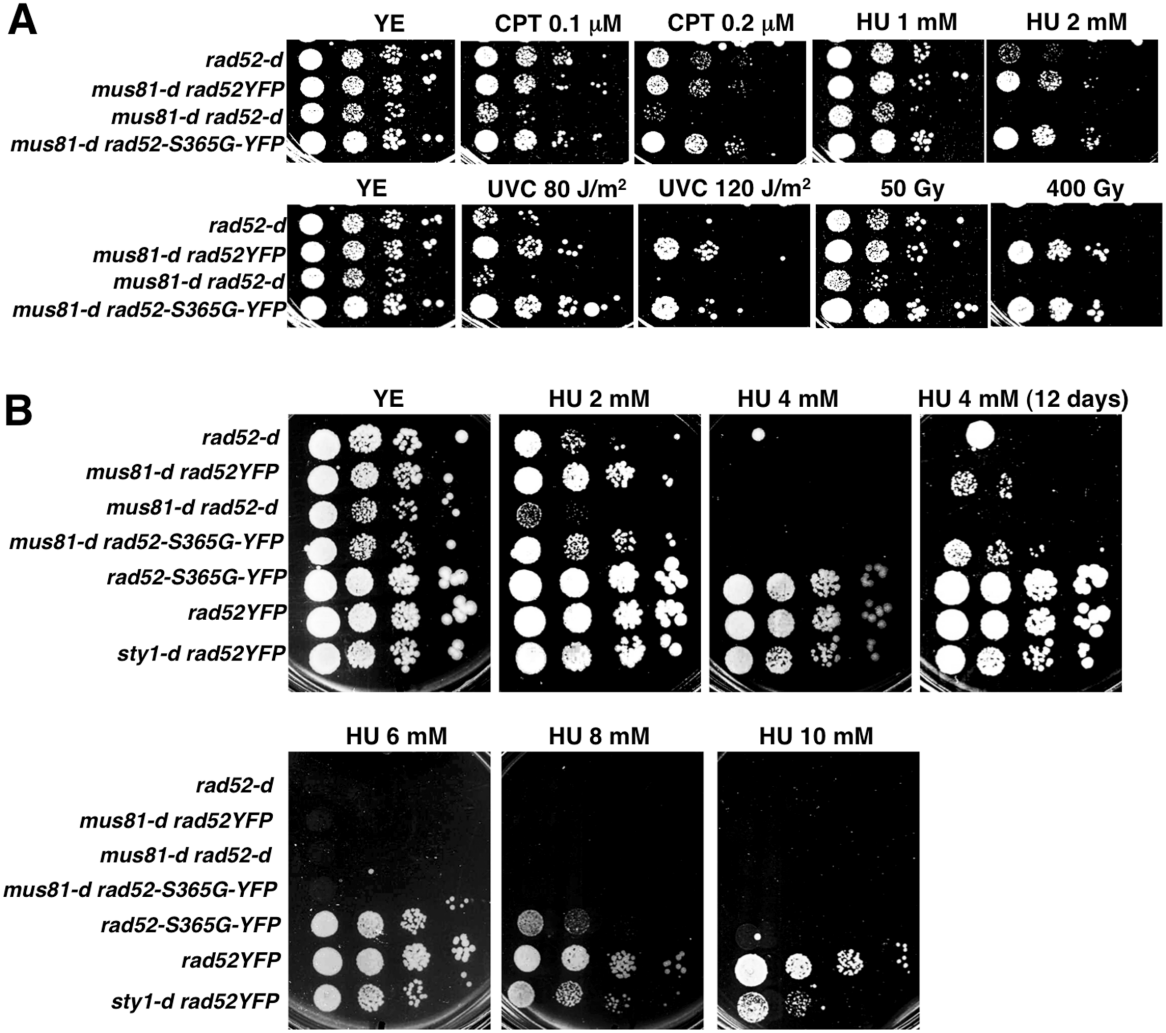
*rad52-S365G-YFP* allele doesn’t change the sensitivity of *mus81-d* cells to genotoxic treatments. Drop assay of the indicated strains on YEc medium. Plates were incubated 5 days at 30°C unless differently specified.

Similar results were obtained when we analyzed the effects of *rad52-S365G-YFP* allele in cells lacking Mus81. Indeed, double mutant *mus81-d rad52-S365G-YFP* behaved similarly to single *mus81-d rad52YFP* mutant even when exposed to HU (Figure 5A and 5B).

Because *rad52-S365G-YFP* mutant is HU sensitive (Figure 3B and 5B) and lacks Rad52 phosphorylation in *rad51-d* and *mus81-d* null cells mimicking *rad51-d sty1-d* and *mus81-d sty1-d* cells (Figure 1A and 1C), we checked the HU sensitivity of *sty1-d* null mutant and found that it is sensitive to HU although to a less extent than *rad52-S365G-YFP* cells (Figure 5B).

### Sty1 Localization is not Affected in *rad51-d* or *mus81-d* Cells

**Figure 6:**
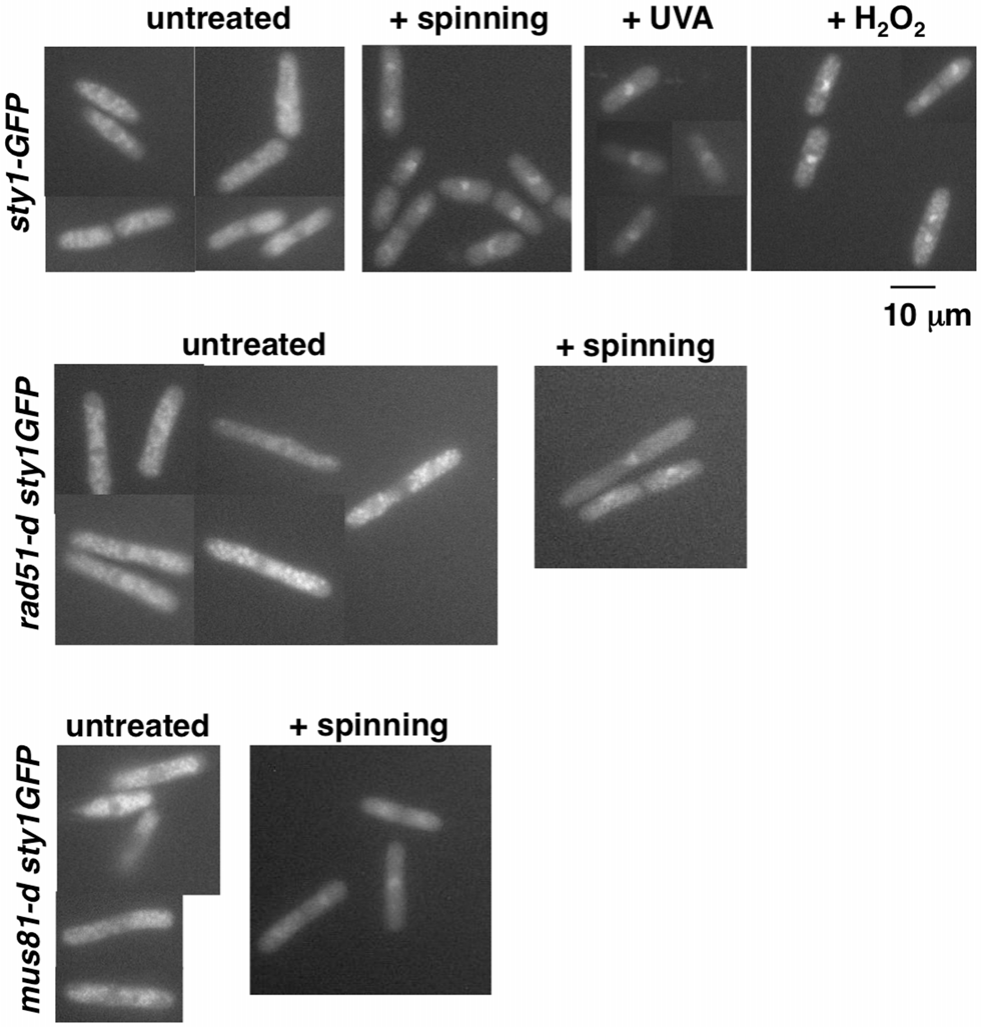
MAP kinase Sty1 localization after stress and in *rad51-d* and *mus81-d* cells. Cells expressing Sty1-GFP from cultures at 5×10^6^ cells/ml were photographed under a fluorescence microscope. As a control, wild type *sty1-GFP* cells were stressed by centrifugation or by exposing to UVA radiation or H_2_O_2_ to monitor stress-induced translocation of Sty1 in the nucleus.

Rad52 is a nuclear protein, while Sty1 kinase resides in the cytoplasm in unstressed cells. Upon stress, activated Sty1 accumulates in the nucleus and after stress adaptation is actively exported to the cytoplasm [37]. Because Rad52 phosphorylation in *rad51-d cells,* and to a lesser extent in *mus81-d* cells, is Sty1 dependent, we asked where Sty1 localizes in these genetic backgrounds, reasoning that may be the absence of the recombinase or of the endonuclease creates a stress that results in Sty1 nuclear retention. To test this hypothesis, we constructed strains deleted for *rad51* or for *mus81* and expressing Sty1-GFP protein. Cells from exponentially growing cultures of *sty1-GFP*, *rad51-d sty1-GFP* and *mus81-d sty1-GFP* strains were analyzed under a fluorescence microscope. As a control, *sty1-GFP* cells were stressed by centrifugation [38] or by exposing cells collected by filtration to UVA or H_2_O_2_ in order to visualize the nuclear retention of Sty1-GFP protein. As shown in Figure 6, Sty1-GFP protein localized in the cytoplasm in unstressed cells and accumulated in the nucleus upon stress for all genetic background tested. Thus, *rad51-d* and *mus81-d* cells display normal Sty1 localization and not massive Sty1 nuclear retention as response to intrinsic stress.

### *rad52-S365G-YFP* Allele Increases the Spontaneous Rate of HR by Rising the Rate of Mus81 Dependent Deletion Type Recombinants

**Figure 7:**
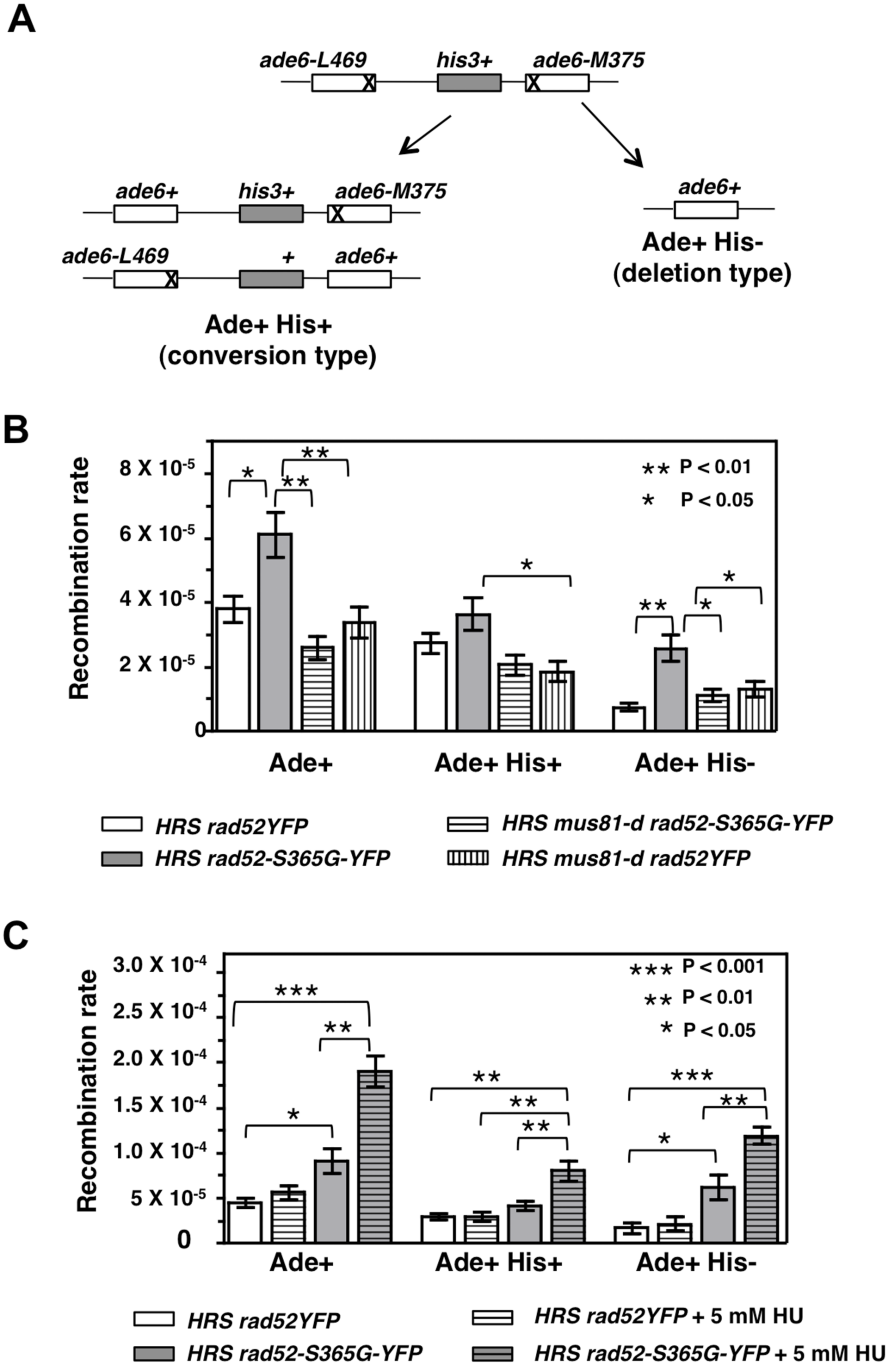
*rad52-S365G-YFP* affects spontaneous recombination rate in a Mus81 dependent manner and displays increased HU induced recombination. (**A**) Representation of the substrate used to estimate spontaneous recombination rate. (**B**) Spontaneous recombination rate at directed repeats of the indicated strains. Bar indicates the mean+/−SEM. ANOVA with Tukey’s test was applied for statistical analysis. (**C**) HU induced recombination rate at directed repeats of the indicated strains. Bar indicates the mean+/− SEM. ANOVA with Tukey’s test was applied for statistical analysis.

The *rad52-S365G-YFP* allele was introduced in cells containing a substrate that allows assessing the rate of spontaneous HR by estimating the frequency of recombination between non-tandem hetero-allelic duplications of the *ade6* gene separated by the *his3^+^* gene [39] hereafter referred to as *HRS* for *h*omologous *r*ecombination *s*ubstrate. With this system, it is possible to calculate the frequency of recombination events issued from gene conversion (Ade+ His+ cells) and the frequency of those leading to deletions of the region between the *ade6* genes (Ade+ His− cells) (Figure 7A). Thus, we assessed the rate of spontaneous HR in cells expressing either wild type Rad52-YFP (*HRS rad52YFP*) or mutant Rad52-S365G-YFP (*HRS rad52-S365G-YFP*) protein by fluctuation test. We found that the mutant strain increased the rate of Ade+ His− recombinants, indicating that mechanisms leading to deletion of the *his3^+^* gene were up regulated in this background. On the opposite, the rate of gene conversion was not different from that of *rad52YFP* control cells (Figure 7B). Because Mus81 plays a major role in the Rad51 independent pathway, we asked if the up-regulated mechanism leading to increased rate of deletion type of recombinants in *rad52-S365G-YFP* cells would require Mus81. For this we performed fluctuation test with strains *HRS mus81-d rad52YFP* and *HRS mus81-d rad52-S365G-YFP*. As shown in figure 7B deleting *mus81* in *rad52-S365G-YFP* cells restored wild type levels of deletion type recombinants.

We also analyzed the effect of *rad52-S365G-YFP* allele on HR induced by replication fork blockage taking advantage of the *RuraR* inducible system described in [40], but we didn’t find any differences between wild type and mutant cells (not shown) indicating that serine 365 phosphorylation does not impact HR mechanisms required to restart blocked replication forks. We also used a reversed mutation assays to evaluate the frequency of deletions and duplications occurring at 20 nucleotides direct repeats flanked by micro-homology [41], but we didn’t find any differences between the two alleles even after growing cells in the presence of sub-lethal concentrations of HU (not shown).

### HU Increases the Rate of Recombinants in *rad52-S365G-YFP* Cells

Because *rad52-S365G-YFP* mutant is HU sensitive, we used the recombination assay to assess the effect of HU on the hyper-recombinant phenotype of *rad52-S365G-YFP* cells. To do so, single Ade− His+ clones were streaked on complete medium supplemented or not with 5 mM HU. Different independents colonies from these plates were resuspended in water and plated for survival estimation and for selection of Ade+ recombinants. As shown in Figure 7C, HU did not affect the recombination rate in wild type cells (*HRS rad52YFP*) while increased the recombination rate of both gene conversion and deletion-type recombinants in *HRS rad52-S365G-YFP* mutant. It is to note that the rate of deletion-type recombinants was three times more than the wild type rate, linking the HU sensitivity of *rad52-S365G-YFP* mutant to increased frequency of loss of genetic material due to unbridled HR.

## Discussion

We previously showed that fission yeast Rad52 protein is phosphorylated upon oxidative stress and in cells lacking Rad51 recombinase and that, in both cases, phosphorylation required the presence of the Sty kinase [30]. Here we report that Rad52 is also phosphorylated in cells lacking the structure-specific endonuclease Mus81 and that phosphorylation is partially dependent on Sty1. In addition, we show that mutating serine 365 in Rad52 results in loss of detectable phosphorylation in cells lacking Rad51 and in partial loss of Rad52 phosphorylation in cells lacking Mus81. On the opposite, the mutation doesn’t change the Rad52 mobility shift after oxidative stress suggesting that Rad52 can exist in different phosphorylation states. This was an unexpected finding since Rad52 phosphorylation upon oxidative stress is dependent on intact Sty1 kinase as for *rad51-d* or *mus81-d* cells ([30] and this work). In addition, analysis of different independent western blots suggest that additional phosphorylations of Rad52 are present in *mus81-d sty1-d rad52YFP* and in *mus81-d rad52-S365G-YFP* cells (Figure 1C), indicating that at least another residue of Rad52 might be modified in *mus81* null cells. That this additional modification might be a phosphorylation is supported by the fact that the **l** phosphatase treatment reverses all the Rad52 slowing migrating bands observed in *mus81-d rad52YFP* cells.

Our findings imply that Sty1 impinges on Rad52 phosphorylation state through different pathways according to the intrinsic perturbation of DNA metabolism or to the extrinsic treatment. In line with this hypothesis, we found that Sty1 kinase localization in *rad51-d* and *mus81-d* cells is similar to that observed in wild-type unstressed cells, meaning that it is mainly in the cytoplasm, while following oxidative stress Sty1 is retained in the nucleus. This different localization of Sty1 might explain its different requirement for Rad52 phosphorylation in the HR mutants versus oxidative stressed cells, especially with regards to serine 365. In agreement with the cytoplasmic localization of Sty1 in *rad51-d* cells, we previously published that in this genetic background cells don’t seem to experience intrinsic oxidative stress [30].

Alternatively, serine 365 might not be the only Rad52 site phosphorylated in cells experiencing oxidative stress and mutating it is not enough to change the mobility shift of the protein. Unfortunately, we were unable to formally prove that Ser365 is phosphorylated *in vivo* in *rad51-d* and *mus81-d* cells and that upon oxidative stress additional sites are phosphorylated in Rad52 since we failed to perform 2D gel electrophoresis followed by mass-spectrometry analysis because of poor focalization of the Rad52YFP protein. However, it remains that Rad52 phosphorylation is not anymore detected in *rad51-d* cells expressing the *rad52-S365G-YFP* allele.

Cells expressing mutant Rad52-S365G-YFP are undistinguishable from wild type cells except when chronically exposed to HU, conditions where they loose viability. This phenotype is not linked to checkpoint failure, but reflects a problem in dealing with chronic replication stress that might lead to accumulation of toxic DNA structures in this specific mutant. Because *rad52-S365G-YFP* cells display an increased frequency of spontaneous deletion-type recombinants issued from mitotic intrachromosomal recombination that are Mus81 dependent, we can speculate that the HU sensitivity is linked to up-regulation of Mus81 dependent pathway required for replication restart when replication forks are perturbed [12] or to uncontrolled Mus81 activity in HR at broken replication forks [13]. In support of this hypothesis, we have shown that HU increases the rate of HR specifically in *rad52-S365G-YFP* cells and that, compared to wild type cells, a three-fold increase in the levels of deletion-type recombinants was observed. Deletion of intervening DNA between repeats can result from different mechanisms including SSA, replication slippage and template exchange between newly synthesized chromatids when replication fork encounters a problem [42], as it has been proposed for example for RNA-DNA hybrids [43].

We have previously published that the rate of spontaneous recombinants in *sty1-d* cells is diminished and that it is the rate of gene conversion that is affected [30]. This differs from the results obtained with the *rad52-S365G-YFP* mutant where an increased frequency of spontaneous deletion-type recombinants was observed. We propose that this discrepancy might reflect the possibility that the Sty1 kinase impinges on different possible targets and not only on Ser365 of Rad52, leading to a different outcome in the HR assay. This is also consistent with the observations that HR dependent recovery of blocked replication forks is affected in *sty1-d* cells [30] but not in the *rad52-S365G-YFP* mutant (not shown), and that *sty1-d* cells are less sensitive to HU than *rad52-S365G-YFP* mutant (Figure 5B).

Although Rad52 phosphorylation is not detected in wild type cells having both Rad51 and Mus81, it might be that transient phosphorylation at serine 365 might occur in these cells in order to favor the Rad51 dependent pathway of HR and prevent error prone pathways of HR. In this hypothesis, Rad52 phosphorylation becomes detectable when the Rad51 dependent pathway of HR is perturbed at the early step of D-loop formation (*rad51-d* cells), or when uncleaved Rad51-independent D-loops accumulate (*mus81-d* cells).

Our study point to a complex post-transcriptional regulation of Rad52 activity in fission yeast, through multiple different phosphorylation events according to the genetic background and to the external insults, that participates in genome stability maintenance. Because Rad52 functions in SSA or Rad51-independent D-loop formation are conserved in the human homologue, we might speculate that the human Rad52 functions in the Rad51 independent pathways of HR might also be controlled through its phosphorylation.
